# Characterization of multiple type-VI secretion system (T6SS) VgrG proteins in the pathogenicity and antibacterial activity of porcine extra-intestinal pathogenic *Escherichia coli*

**DOI:** 10.1080/21505594.2019.1573491

**Published:** 2019-01-24

**Authors:** Bingbing Zong, Yanyan Zhang, Xiangru Wang, Manli Liu, Tongchao Zhang, Yongwei Zhu, Yucheng Zheng, Linlin Hu, Pei Li, Huanchun Chen, Chen Tan

**Affiliations:** a State Key Laboratory of Agricultural Microbiology, College of Veterinary Medicine, Huazhong Agricultural University, Wuhan, Hubei, China; b Key Laboratory of Preventive Veterinary Medicine in Hubei Province, The Cooperative Innovation Center for Sustainable Pig Production, Wuhan, Hubei, China; c Key Laboratory of Development of Veterinary Diagnostic Products, Ministry of Agriculture of the People’s Republic of China, Wuhan, Hubei, China; d International Research Center for Animal Disease, Ministry of Science and Technology of the People’s Republic of China, Wuhan, Hubei, China; e Hubei Biopesticide Engineering Research Centre, Hubei Academy of Agricultural Sciences, Wuhan Hubei, China

**Keywords:** Porcine ExPEC PCN033, antibacterial activity, pathogenicity, T6SS, VgrG, gene deletion

## Abstract

Porcine extra-intestinal pathogenic *Escherichia coli* (ExPEC) causes great economic losses to the pig industry and poses a serious threat to public health worldwide. Some secreted virulence factors have been reported to be involved in the pathogenicity of the infection caused by ExPEC. Type-VI secretion system (T6SS) is discovered in many Gram-negative bacteria and contributes to the virulence of pathogenic bacteria. Valine-glycine repeat protein G (VgrG) has been reported as an important component of the functional T6SS. In our previous studies, a functional T6SS was identified in porcine ExPEC strain PCN033. Further analysis of the PCN033 genome identified two putative *vgrG*s genes (*vgrG1* and *0248*) located inside T6SS cluster and another two (*vgrG2* and *1588*) outside it. This study determined the function of the four putative VgrG proteins by constructing a series of mutants and complemented strains. *In vitro*, the VgrG1 protein was observed to be involved in the antibacterial ability and the interactions with cells. The animal model experiment showed that the deletion of *vgrG1* significantly led to the decrease in the multiplication capacity of PCN033. However, the deletion of *0248* and/or the deletion of *vgrG2* and *1588* had no effect on the pathogenicity of PCN033. The study of four putative VgrGs in PCN033 indicated that only VgrG1 plays an important role in the interaction between PCN033 and other bacteria or host cells. This study can provide a novel perspective to the pathogenesis of PCN033 and lay the foundation for discovering potential T6SS effectors.

## Introduction

Extra-intestinal pathogenic *Escherichia coli* (ExPEC) can infect the tissues of the distal intestinal tract and cause various diseases in humans and animals [1–3]. ExPEC includes uropathogenic *E. coli* (UPEC), neonatal meningitis-causing *E. coli* (NMEC), avian pathogenic *E. coli* (APEC), and septicemic *E.coli* (SEPEC) [4–6]. ExPEC usually lacks pathogenicity when it is colonized in the intestine. But when these pathogens migrate to extra-intestinal organs, they can cause various life-threatening diseases such as urinary tract infections, newborn meningitis, peritonitis, bacteremia, and septicemia [4,5,7–9]. ExPEC has caused a high mortality and economic losses in swine industry so far. It has posed a serious threat to human health and increased animal industry costs worldwide [5,10,11].

With the rapid development of the swine industry in China, the growth trend of the outbreak of swine diseases caused by ExPEC has become an urgent issue [12]. Porcine ExPEC is an important pathogen causing meningitis, pneumonia, arthritis, and septicemia and is highly resistant to multiple drugs [12–15]. Moreover, some similar virulence profiles and serogroups have been reported to be found in both porcine and human ExPEC, suggesting that there is a cross-infection potential between human and pigs [12,16,17]. However, the pathogenic mechanism of porcine ExPEC remains poorly understood. Therefore, it is necessary to study the pathogenesis of porcine ExPEC so as to more effectively prevent the disease caused by ExPEC and facilitate the rapid development of swine industry and the improvement of human health. In one of our previous studies, a virulent porcine ExPEC strain PCN033 was isolated from the brain of a diseased pig and its whole genome was sequenced [18]. Subsequently, an integrated T6SS which plays an important role in the pathogenicity of PCN033 was identified [18,19]. However, the mechanism of T6SS involved in PCN033 infection remains unclear.

As an important virulence factor, T6SS plays a key role in microbial competition and bacterial infection [20–23]. It has taken ten years for T6SS to be named ever since it was first discovered. Williams et al. [24] firstly identified Hcp (hemolysin coregulated protein) and proposed that it traversed the outer membrane via a “novel mechanism of secretion”. Subsequently, Wang et al. [25] found the link between *rhs* (recombination hotspot) and *hcp* in *V.cholerae*. Das and Chaudhuri [26] named these *rhs* elements IAHPs (IcmF associated homologous proteins) and they speculated that IAHPs were likely to encode a secretion apparatus. Rao et al. [27] provided both genetic and biochemical evidence that IAHPs encoded a new type of secretion. This protein secretion pathway was defined as T6SS in *Vibrio cholerae* and was visualized in *Pseudomonas aeruginosa* in 2006 [28,29]. Subsequent studies reported the presence of T6SS in many bacteria and its contribution to the antibacterial activity, colonization, and virulence [30–35]. Although the precise structure of T6SS has not been successfully resolved, it was reported to be homologous to bacteriophage tail structures [36,37]. Previous study has revealed that an integrated and functional T6SS consisted of at least 13 conserved components [38]. These conserved components of T6SS assembles into trans-envelope complex, inner tube, puncturing needle/spike, tail tube/sheath, and baseplate [39,40]. The trans-envelope complex of T6SS constitutes of the TssJ, TssL, TssM and TssJLM was used as a docking station [39,41–46]. The inner tube consistes of the hexameric Hcp rings tipped by the trimeric VgrG-PAAR puncturing device and the tail tube/sheath consistes of TssB/C subunits [33,47–50]. The baseplate is composed of TssE, TssF, TssG, TssK, and VgrG27 [51]. In addition, ClpV provides energy for the activity of T6SS and depolymerizing the TssB/TssC (VipA/VipB) for recycling and reassembly [33,52]. VgrG was reported to be an important core component of T6SS and to have a trimeric structure comprising the tip of the nanotube [28,37,40,50,51,53]. VgrG contains two domains which are homologous to the proteins constituting the bacteriophage tail (namely, gp5 and gp27) [50]. VgrG-PAAR presumably functions to penetrate the prey cell with VgrG’s needle-shaped C-terminal β-helical domain and its C-terminal domains acts as effectors and/or bound effectors [36,54–58]. Some reports showed that the deletion of *vgrG* prevented the formation of integrated T6SS, resulting in the failure of effector protein to be secreted into extracellular environment [51,53,59,60]. Moreover, VgrG was reported to be crucial to the pathogenesis of some pathogens [61,62]. In addition, multiple *vgrG* genes are found in many bacteria encoding T6SS [36,51], however, whether all of them play an important role is still unclear.

Functional VgrGs possessing a complete gp27/gp5 domain have widely been reported [36,50]. Although some proteins have no gp27/gp5 domain, they are also annotated as VgrGs by the NCBI Prokaryotic Genome Annotation Pipeline (https://www.ncbi.nlm.nih.gov/genome/annotation_prok/). However, these VgrGs possessing no conserved gp27/gp5 domain have scarcely been studied. This study covered the four putative VgrGs with a complete gp27/gp5 domain or without gp27/gp25 domain. Two putative *vgrG*s (*0247* and *0248*) located inside main T6SS gene cluster and another two putative *vgrG*s (*1587* and *1588*) located outside main T6SS gene cluster in the whole genome of PCN033 were identified. However, their functions remain unknown. Therefore, this study has investigated the function of the four putative VgrG proteins with and without gp27/gp5 domain in PCN033 by knocking out these *vgrG* genes. These mutants will be used to determine the function of the four putative VgrGs by investigating the changes in virulence of PCN033. This study is aimed to lay the foundation for discovering potential T6SS effectors and develop the effective antibacterial drugs to treat porcine ExPEC diseases.

## Results

### The location of four VgrG genes in PCN033

**Figure 1.**
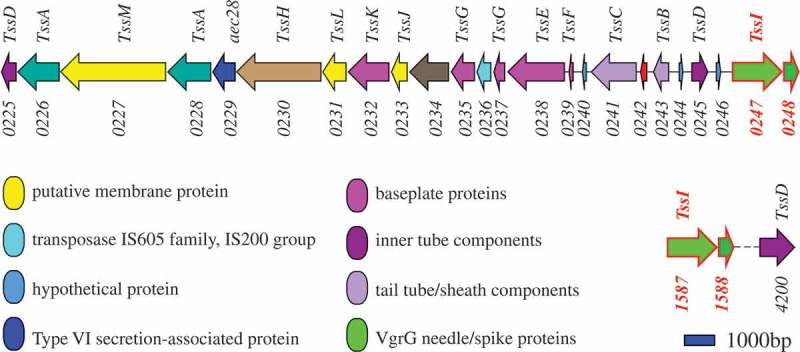
Genetic map of T6SS-related (type 6 secretion system) genes in PCN033. The corresponding components of genes to T6SS are labeled above. The locus tags of T6SS-related genes in PCN033 are marked below. Genes *vgrG1* (PCN033_0247) and *0248* (PCN033_0248) are present in the T6SS cluster, and genes *vgrG2* (PCN033_1587) and *1588* (PCN033_1588) are located outside the T6SS cluster. Sequencing data for PCN033 can be obtained from the National Centre for Biotechnology Information (NZ-CP006632.1).

Hcp and VgrG are considered as the hallmarks of T6SS [29,63,64]. VgrG is a needle-like structure of T6SS and is homology to the T4 bacteriophage cell-puncturing device [36,65]. Hcp protein forms homohexameric rings with an inner diameter of 40 Å and acts as tube components of T6SS, allowing effectors to pass through the tube center [29]. One T6SS cluster containing 24 encoding sequences and three T6SS-related genes (*vgrG2, 1588, hcp*3) outside the T6SS cluster were identified in PCN033. The genes *vgrG1* and *0248* were found to be located in the T6SS cluster. The T6SS-related genes *vgrG2* and *1588* were located to be far away from the T6SS cluster (Figure 1).

### The homology and the gp27/gp5 domains labeling of VgrGs in PCN033

**Figure 2.**
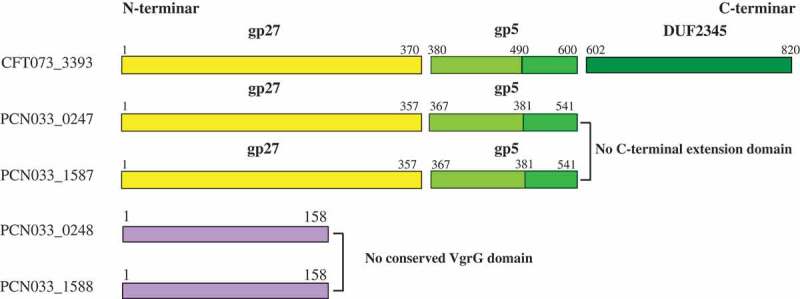
Conserved domain labeling of four putative VgrGs in PCN033. The gp27/gp5 domains of VgrGs of PCN033 were labeled according to the VgrG of CFT073. Proteins are labeled with their respective gene numbers on the left. The gp27/gp5 domain and the residue number are shown above. Different domains are marked in distinct colors. The gp27 domain is colored yellow and the gp5 domain is colored light green and green. The light green part indicates the gp5 OB fold domain and the DUF2345 domain is colored dark green color and purple represents the absence of conserved domain.

The amino acid sequences of 0247 and 1587 were found to be 99% homologous (both 541 aa) and the amino acid sequences of 0248 and 1588 were identical (both 158 aa) (Figure S1(a)). The amino acid sequences of these four putative VgrGs were also analyzed with NCBI Conserved Domain Search. The results showed that 0247 and 1587 possessed the conserved VgrG domain (named as VgrG1 and VgrG2, respectively), while 0248 and 1588 had no conserved VgrG domain (expressed by their locus number tentatively) (Figure S1(b)). Further analysis found that VgrG1 and VgrG2 of PCN033 were approximately 25% homologous to the N-terminus (residues 1–597) of *Escherichia coli* CFT073 VgrG (Accession number: GCA_000007445.1) (Figure S1(c)). The gp27/gp5 domains of VgrGs in PCN033 were labeled according to the VgrG of CFT073, based on the analysis of Clustal Omega and conserved domain (Figure 2).

### Contribution of VgrG to the virulence and antibacterial activity of PCN033

**Figure 3.**
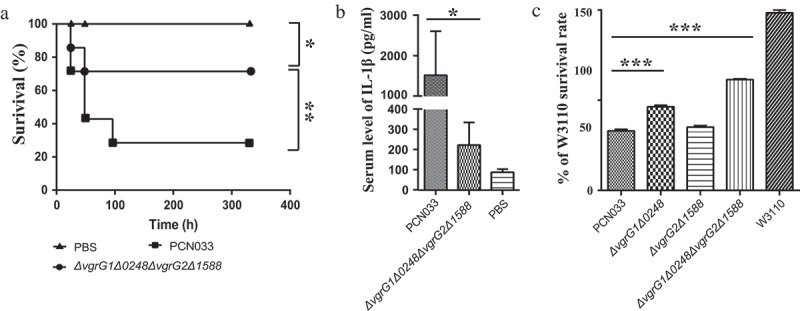
Roles of VgrG family proteins in the virulence and antibacterial activity of PCN033. (a) Survival rate of mice infected with PCN033 or Δ*vgrG1*Δ*0248*Δ*vgrG2*Δ*1588*. (b) Serum IL-1β levels in mice infected with PCN033 and mutant Δ*vgrG1*Δ*0248*Δ*vgrG2*Δ*1588*. (c) The antibacterial ability of PCN033, Δ*vgrG1*Δ*0248*, Δ*vgrG2*Δ*1588*, and Δ*vgrG1*Δ*0248*Δ*vgrG2*Δ*1588*. Means and SD of three independent experiments in triplicate are calculated. Significant differences between groups are indicated: *(*P* < 0.05), **(*P* < 0.01) and ***(*P* < 0.001).

To determine whether the deletion of *vgrG* impairs the virulence of PCN033, female BALB/c mice (5 weeks old) were inoculated intraperitoneally (i.p.) with approximate 1 × 10^6^ CFU of Δ*vgrG1*Δ*0248*Δ*vgrG2*Δ*1588*, PCN033, or PBS. After infection for 336 hours (14 days), the survival rate of mice infected with Δ*vgrG1*Δ*0248*Δ*vgrG2*Δ*1588* mutant was significantly higher than that of mice infected with PCN033 (Figure 3(a), ** P < 0.01). Inflammation plays an important role in bacterial infection [66]. Thus, we compared the abilities of the strains to induce inflammation by measuring the production of serum IL-1β protein. The level of IL-1β in mice infected with mutant Δ*vgrG1*Δ*0248*Δ*vgrG2*Δ*1588* was significantly lower than that in mice infected with PCN033 (Figure 3(b), * P < 0.05), suggesting that VgrG played a major role during the process of inflammation in PCN033. These results indicated that VgrGs were involved in the virulence of PCN033 in mice.

To investigate the role of VgrG in the antibacterial process, the antibacterial ability of mutants Δ*vgrG1*Δ*0248*, Δ*vgrG2*Δ*1588*, and Δ*vgrG1*Δ*0248*Δ*vgrG2*Δ*1588* was compared with that of PCN033. In comparison of the amount of W3110 in different groups, higher amount indicated weaker antibacterial ability, while lower amount of W3110 indicated antibacterial advantages of predator bacteria. The results showed that the antibacterial ability of mutants Δ*vgrG1*Δ*0248* and Δ*vgrG1*Δ*0248*Δ*vgrG2*Δ*1588* was significantly lower than that of PCN033, whereas the antibacterial ability of mutant Δ*vgrG2*Δ*1588* did not alter compared with that of PCN033 (Figure 3(c), *** P < 0.001). The analysis showed that VgrG1 and/or 0248 in T6SS was involved in the antibacterial process of PCN033.

### Effects of VgrG in T6SS on the interactions between PCN033 and HBMEC cells

**Figure 4.**
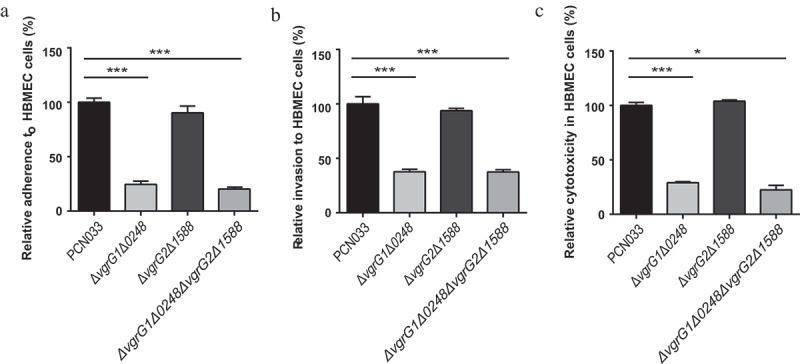
Interaction between bacteria and HBMEC cells. A, B, and C respectively indicate adherence ability, invasion ability, and the cytotoxicity of parental and mutant strains to HBMEC cells. Rates of adherence, invasion and cytotoxicity are expressed relative to those of WT PCN033 (100%). Means and SD of three independent experiments in triplicate are calculated. Significant differences between groups are indicated: *(*P* < 0.05) and ***(*P* < 0.001).

To investigate whether VgrG participated in the interactions between PCN033 and HBMEC (Human Brain Microvascular Endothelial Cells), the abilities of mutants Δ*vgrG1*Δ*0248*, Δ*vgrG2*Δ*1588*, and Δ*vgrG1*Δ*0248*Δ*vgrG2*Δ*1588* to adhere to and invade HBMEC cells were compared with those of the parental strain. The experiment results revealed that the adherence and invasion abilities of the mutants Δ*vgrG1*Δ*0248* and Δ*vgrG1*Δ*0248*Δ*vgrG2*Δ*1588* to HBMEC cells were significantly lower than those of PCN033 and mutant Δ*vgrG2*Δ*1588* to HBMEC cells (Figure 4(a, b), *** P < 0.001). Moreover, the cytotoxic activities of mutants Δ*vgrG1*Δ*0248* and Δ*vgrG1*Δ*0248*Δ*vgrG2*Δ*1588* to HBMEC cells were significantly lower than those of PCN033 and mutant Δ*vgrG2*Δ*1588* to HBMEC cells (Figure 4(c), * P < 0.05, *** P < 0.001). These results indicated that VgrG1 and/or 0248 in T6SS mediated the interaction between PCN033 and HBMEC cells, which was consistent with the antibacterial experiment results.

### Contribution of VgrG1 to the multiplication capacity of PCN033 in mice

**Figure 5.**
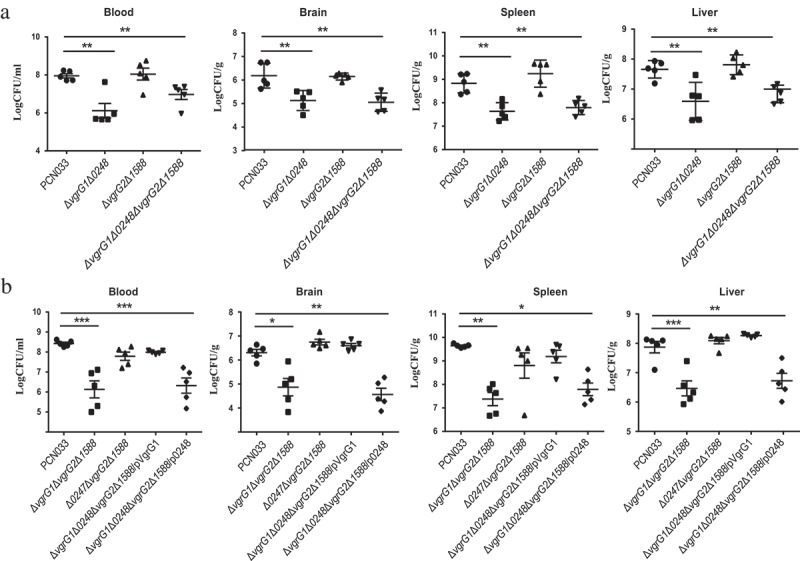
Contribution of VgrG1 in T6SS to the multiplication capacity of PCN033 in mice. (a) Multiplication capacity of parental and mutant strains in different tissues of mice. (b) Multiplication ability of parental, mutants, and complemented strains in different tissues of mice. Significant differences between groups are indicated: *(*P* < 0.05), **(*P* < 0.01) and ***(*P* < 0.001).

To determine that the effect of VgrG on the multiplication capacity of PCN033 in the host, the multiplication capacities of bacteria in mice were investigated. Female BALB/c mice (5 weeks old) were inoculated intravenously (i.v.) with approximate 1 × 10^7^ CFU of Δ*vgrG1*Δ*0248*, Δ*vgrG2*Δ*1588*, Δ*vgrG1*Δ*0248*Δ*vgrG2*Δ*1588*, PCN033, or PBS. Bacterial counts in the blood and organs of mice were determined at 6 h post-infection. Bacterial counts in the blood, brain, liver, and spleen of mice infected with mutants Δ*vgrG1*Δ*0248* and Δ*vgrG1*Δ*0248*Δ*vgrG2*Δ*1588* were significantly reduced compared with those of mice infected with PCN033 (Figure 5(a), ** P < 0.01), while no significant difference in the bacterial counts was observed in mice infected with mutant Δ*vgrG2*Δ*1588* and with PCN033. In addition, the multiplication capacities of mutants Δ*vgrG1*Δ*vgrG2*Δ*1588* and Δ*0248*Δ*vgrG2*Δ*1588* were also measured. Consistent with the above-mentioned results, the multiplication capacity of mutant Δ*vgrG1*Δ*vgrG2*Δ*1588* was significantly lower than that of PCN033, while the multiplication capacity of mutant Δ*0248*Δ*vgrG2*Δ*1588* showed no difference from that of PCN033 (Figure 5(b), * P < 0.05, ** P < 0.01, *** P < 0.001). Complementation of VgrG1 but not 0248 is able to restore the multiplication capacity to that of wild type. These results suggested that VgrG1 but not 0248 involved in the multiplication of PCN033 in the blood and organs, and that VgrG1 played a pivotal role in the multiplication process.

### Survival of PCN033 in pig blood facilitated by VgrG1 in T6SS

**Figure 6.**
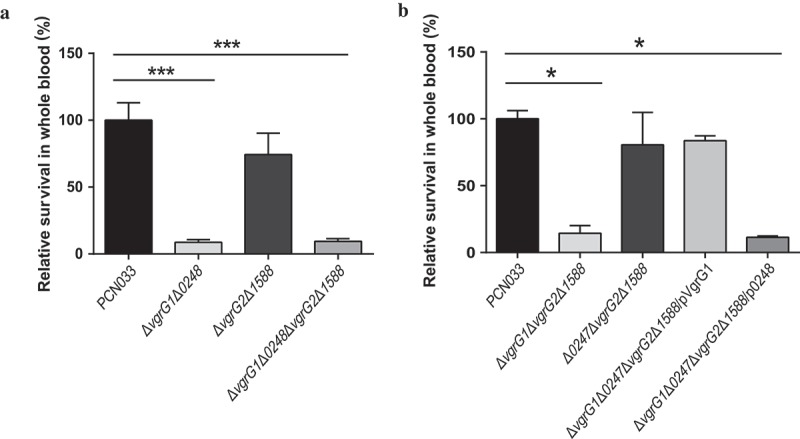
Viability of parental, mutant and corresponding complemented strains in pig whole blood. (a) Survival rate of parental and mutant strains in pig whole blood. (b) Survival rate of parental, mutants and complemented strains in pig whole blood. The survival rates are expressed relative to those of WT PCN033 (100%). Means and SD of three independent experiments in triplicate are calculated. Significant differences between groups are indicated: *(*P* < 0.05) and ***(*P* < 0.001).

To evaluate the role of VgrG in evasion of innate immune responses, we measured the survival rate of PCN033, *vgrG* mutants, and complemented strains in whole blood collected from pigs. The experiment results showed that the survival rate of mutants Δ*vgrG1*Δ*0248* and Δ*vgrG1*Δ*0248*Δ*vgrG2*Δ*1588* was significantly lower than that of PCN033 (Figure 6(a), *** P < 0.001). Simultaneously, the comparison of Δ*vgrG1*Δ*vgrG2*Δ*1588* and Δ*0248*Δ*vgrG2*Δ*1588* mutants with PCN033 revealed that the survival rate of Δ*vgrG1*Δ*vgrG2*Δ*1588* was significantly lower than that of PCN033, whereas no difference in survival rate was found between mutant Δ*0248*Δ*vgrG2*Δ*1588* and PCN033 (Figure 6(b), * P < 0.05). Complementation of VgrG1 but not 0248 is able to restore the survival capacity in pig whole blood to that of wild type. These results suggested that VgrG1 but not 0248 participated in immune evasion of PCN033, and that VgrG1 played a pivotal role in evasion of innate immune responses in the pig whole blood.

### Functional difference between proteins VgrG1 and VgrG2 in PCN033

**Figure 7.**
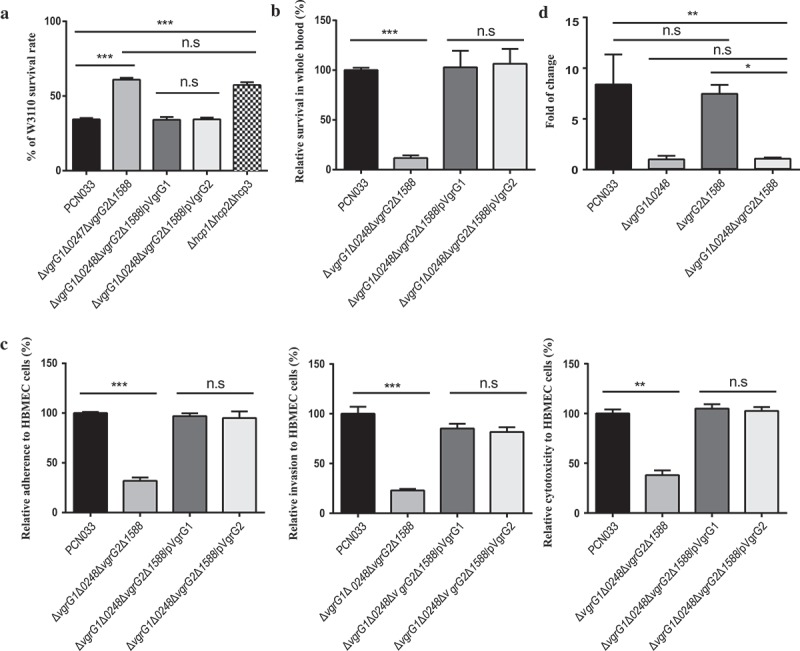
Different functions between VgrG1 and VgrG2 and effect of VgrG1 on activity of T6SS. (a) Antibacterial assay of PCN033, Δ*vgrG1*Δ*0248*Δ*vgrG2*Δ*1588*, Δ*vgrG1*Δ*0248*Δ*vgrG2*Δ*1588*/pVgrG1, Δ*vgrG1*Δ*0248*Δ*vgrG2*Δ*1588*/pVgrG2, and Δ*hcp1*Δ*hcp2*Δ*hcp3*. (b) Survival rate of strains in pig blood. The survival rate is expressed relative to that of WT PCN033 (100%). (c) Rates of adherence and invasion and cytotoxicity of strains to HBMEC cells. Rates of adherence, invasion and cytotoxicity to HBMEC cells are expressed relative to those of WT PCN033 (100%). (d) The transcription level of VgrG1 and VgrG2. Means and SD of three independent experiments in triplicate are calculated. Significant differences between groups are indicated: *(*P* < 0.05), **(*P* < 0.01) and ***(*P* < 0.001).

Interestingly, we found that VgrG1 and VgrG2 only differed in one amino acid, but mutant studies indicated that only deletion of *vgrG*1 but not *vgrG2* exhibited the phenotypes in both interbacterial competition and virulence. To further examine the functions of VgrG1 and VgrG2, we constructed a complemented strain Δ*vgrG1*Δ*0248*Δ*vgrG2*Δ*1588*/pVgrG2 (Figure S3(d)) and measured its growth rate as described above (Figure S5). And then, the antibacterial activity and survival rate in the pig whole blood of Δ*vgrG1*Δ*0248*Δ*vgrG2*Δ*1588*/pVgrG2, and its interaction with HBMEC were compared with those of WT, Δ*vgrG1*Δ*0248*Δ*vgrG2*Δ*1588*, and Δ*vgrG1*Δ*0248*Δ*vgrG2*Δ*1588*/pVgrG1. Significant differences were found only between WT group and Δ*vgrG1*Δ*0248*Δ*vgrG2*Δ*1588* group. However, trans complementation of either VgrG1 or VgrG2 in *vgrG*s mutants is able to restore both the antibacterial and virulence phenotypes (Figure 7(a–c), ** P < 0.01, *** P < 0.001). The quantitative experiments found that VgrG2 was not expressed under our experimental conditions (Figure 7(d), * P < 0.05, ** P < 0.01). In general, these findings indicated that the difference in one amino acid did not affect the functions of proteins VgrG1 and VgrG2. The lack of *vgrG2* mutant phenotype may be due to little expression of endogenous expression of VgrG2 but overexpression on the plasmid is able to exhibit its function similar to VgrG1.

### Contribution of VgrG to T6SS in PCN033

To further investigate whether VgrG impaired T6SS activity completely or partly, the antibacterial ability of mutants Δ*vgrG1*Δ*0248*Δ*vgrG2*Δ*1588* and Δ*hcp1*Δ*hcp2*Δ*hcp3* was compared with that of PCN033. The results showed that the antibacterial ability of mutants Δ*vgrG1*Δ*0248*Δ*vgrG2*Δ*1588*, Δ*hcp1*Δ*hcp2*Δ*hcp3* was significantly lower than that of PCN033, and that no significant difference in antibacterial ability was found between mutant Δ*vgrG1*Δ*0248*Δ*vgrG2*Δ*1588* and Δ*hcp1*Δ*hcp2*Δ*hcp3* (Figure 7(a), *** P < 0.001), suggesting that the effect of the deletion of *vgrG* on the antibacterial activity of PCN033 was consistent with that of the deletion of *hcp*. Since the secretion of Hcp was reported to be the hallmark of a functional T6SS [29,30,36], it could be concluded that the deletion of VgrG might impair the whole activity of T6SS by affecting the assembly of T6SS in PCN033.

## Discussion

ExPEC-related diseases seriously affect human health and meat industry, resulting in substantial economic losses [5,10,11,67]. The infection rate of porcine ExPEC in Chinese farms has gradually increased, and 81.9%-100% of ExPEC isolates exhibit broad-spectrum drug resistance [12,14]. A series of virulence factors of ExPEC including secreted toxins, adhesions, iron acquisition systems (siderophores), and capsular antigens have been reported [5,68]. Recent studies have reported that T6SS plays an important role in the competition and pathogenicity of ExPEC [69–71].

T6SS widely occurs in approximate 25% of all sequenced Gram-negative bacteria, including the members of the genera *Vibrio, Pseudomonas, Burkholderia, Serratia, Edwardsiella*, and *Enterobacter* [28–32,70,71]. T6SS contributes to the pathogenicity, competition, proliferation, and cooperation [30,72–74]. T6SS is structurally, functionally, and evolutionarily related to the contractile injection systems (CIS), a broad family of machines with a spring-like mechanism to deliver macromolecules into target cells [75–78]. Our previous studies found that porcine ExPEC caused serious diseases in China. Our comparative genomic analyses indicated that an integrated T6SS gene cluster was present in the virulent strain PCN033, whereas it was absent in the low pathogenic strain PCN061 [12,18]. The transport process of T6SS is similar to the infection process of T4 phage. When the baseplate complex anchors to the cell membrane, the VipA/VipB sheath is contracted, and the HCP tube and VgrG needle-like complex are injected into host cells [37,50]. VgrG, an important component of T6SS, is homologous to the T4 phage gp27/gp5 complex [30,50,64,79]. It is predicted that VgrG represents the central part of the baseplate and acts as the tail tube-puncturing device connector [35,51,53,59,60]. In this study, four putative VgrGs were found to be encoded in PCN033. Further sequence analysis showed that VgrG1 and VgrG2 possessed a complete gp27/gp5 domain. It should be noted that although 0248 and 1588 were found to be homologous to VgrG by BLASTP, 0248 and 1588 did not possess a conserved VgrG domain. Thus, we speculated that VgrG1 and VgrG2 were involved the function of T6SS, while whether 0248 and 1588 participated in the function of T6SS remained unclear. To test our hypothesis and to characterize the function of all four putative VgrGs in PCN033, a series of experiments were carried out.

T6SS is a key virulence factor for some pathogenic bacteria and its function is closely related to bacterial pathogenesis [53,80]. To investigate the effect of VgrGs on the pathogenicity of PCN033, the survival rate of mice was compared. The results showed that the deletion of *vgrG*s significantly weakened the virulence of PCN033 (Figure 3(a)). T6SS was reported to have activated pyrin inflammasome and triggered inflammation in the recent reports [72,81]. To further study the role of VgrG proteins in PCN033 pathogenesis, the serum level of IL-1β in mice was detected. We drew that the level of IL-1β was significantly reduced after the deletion of *vgrG*s (Figure 3(b)). The findings above suggested that VgrG proteins of T6SS in PCN033 played an important role in the pathogenicity of PCN033, which was consistent with previous reports [28,29,70,72].

The ability of antibacterial was reported to be vital for the survival and pathogenicity of *Pseudomonas aeruginosa* and *enteroaggregative E. coli* [30,71]. The antibacterial activity of T6SS is beneficial to the access to the niche, nutrients, or DNA. In most cases, T6SS causes the damage to bacterial cells of competitor [82,83]. In order to reveal the functions of VgrGs both inside and outside the T6SS cluster, further exploration was performed. The antibacterial experiment results showed that only genes *vgrG1* and *0248* contributed to the antibacterial ability of PCN033 (Figure 3(c)). Bacterial adherence to and interaction with epithelial cells are prerequisites for the induction of bacteria infection [63,84]. Similarly, only genes *vgrG1* and *0248* effected the adherence and invasion abilities of PCN033 (Figure 4(a, b)), which was consistent with the above results. In addition, previous studies reported that VgrG in *Vibrio cholerae* and *Aeromonas hydrophila* induced cell toxicity [85,86]. Therefore, the cytotoxicity of PCN033 and mutants was compared, the results were consistent with the results obtained from the examination of antibacterial ability, adherence and invasion (Figure 4(c)). Therefore, it could be concluded that only VgrG1 and 0248 located inside the T6SS serve a function in PCN033 pathogenicity, which is consistent with the hypothesis that VgrG1 is functional. However, the role of 0248 remains to be further explored.

In order to further identify the function of VgrG1 and 0248 proteins of T6SS in PCN033, the mutants Δ*vgrG1*Δ*vgrG2*Δ*1588*, Δ*0248*Δ*vgrG2*Δ*1588* and the complemented strain Δ*vgrG1*Δ*0248*Δ*vgrG2*Δ*1588*/pVgrG1, Δ*vgrG1*Δ*0248*Δ*vgrG2*Δ*1588*/p0248 were constructed (Figure S3). Hood et al. [30] and Schell et al. [87] reported that the absence of T6SS significantly decreased the multiplication capacity of bacteria in host. Consistent with their results, this study found that multiplication capacity was significantly decreased in blood, brain, spleen and liver after the deletion of *vgrG1* (Figure 5(a, b)). Furthermore, mutant Δ*vgrG1*Δ*0248*Δ*vgrG2*Δ*1588* complemented with *vgrG1* but not *0248* could recover its multiplication capacity in host. (Figure 5(b)). To further confirm the conclusion above, the survival rate in porcine whole blood was examined since PCN033 was isolated from the brain of a diseased pig. As was observed above, only VgrG1 played a role in the survival of PCN033 in porcine whole blood (Figure 6(a, b)). Based on these findings, it could be concluded that only VgrG1 inside T6SS plays an important role in the pathogenic process of PCN033, while proteins 0248, VgrG2 and 1588 are less important for PCN033 virulence.

The reason why 0248 and 1588 have no function could be attributed to the fact that they have no conserved VgrG domain (Figure 2 and Figure S1(b)). Although VgrG1 and VgrG2 are 99% homologous and they have complete conserved VgrG gp27/gp5 domain (Figure S1(a, b) and Figure 2), they have different functionality in PCN033. Considering this, we speculate that the difference in function between VgrG1 and VgrG2 in PCN033 may be due to amino acid mutations or their different expressions in PCN033. In the latest report, VgrG gp27-like hube domain is sufficient for the assembly and the mechanism of action of the T6SS [51]. Therefore, the different functions of VgrG1 and VgrG2 in PCN033 are most likely to result from their different expressions in PCN033. In the further experiments, we found that the functionality of Δ*vgrG1*Δ*0248*Δ*vgrG2*Δ*1588* was restored when VgrG1 was complemented, so was VgrG2 (Figure 7(a–c)). Moreover, the quantitative experiments found that VgrG2 was not expressed in PCN033 under our experimental conditions (Figure 7(d)). Therefore, the difference in one amino acid might not have affected the function of proteins VgrG1 and VgrG2. The difference in functions between VgrG1 and VgrG2 in PCN033 are most likely to be attributed to their different expressions.

It was reported that VgrG affected the pathogenicity and antibacterial activity of T6SS as a structure component or effector (evolved VgrGs) [21–23,57,58]. The evolved VgrG proteins possess a C-terminal extension domain as an extra effector domains, for example, the actin cross-linking domain of VgrG-1 (antieukaryotic) and peptidoglycan hydrolase domain of VgrG-3 (antibacterial) in *V. cholerae* [54,85]. Our results showed that VgrG1 had the gp27/gp5 domain, but it did not contain the C-terminal extension domain (Figure 2). Therefore, it could concluded that VgrG1 might have effect on the activity of T6SS by affecting the normal assembly of T6SS. This is consistent with the recent reports that VgrG could polymerize the TssEFGK wedges to form a baseplate and could be attached to the cell membrane and recruit Hcp hexamers to facilitate assembly of T6SS [43,51]. In our previous study, we found that Hcp proteins involved in the antibacterial activity and virulence of PCN033 [19]. In the further experiments, we found that the effect of deletion of *vgrG*s on the antibacterial activity of PCN033 was consistent with that of deletion of *hcp*s (Figure 7(a)). Therefore, it could be further concluded that VgrG1 could affecting the normal assembly of T6SS and result in the decrease in pathogenicity of PCN033.

T6SS is a versatile protein export machine that can directly deliver toxins into eukaryotic cells as well as other bacteria [20–23,30,31,40]. Recently, variety of effectors secreted by T6SS have been identified and the majority of them are antibacterial toxins [23,71], including peptidoglycan amidase, peptidoglycan glycoside hydrolase, phospholipase, and several nuclease enzymes [22,23,71]. By sequence alignment, we found some potential effectors of T6SS in PCN033 (Table S2), such as, lysozyme-like protein (PCN033_0240), RHS element proteins (PCN033_0249, PCN033_0251, and PCN033_0252), and hydrolase (PCN033_0256 and PCN033_0262), etc. Therefore, we speculate that these potential effectors delivered by T6SS may directly contribute to the antibacterial activity and the virulence of PCN033. VgrG1 could affect the normal assembly of T6SS and prevent the secretion of the toxin effectors and ultimately contribute to the pathogenicity of PCN033.

Collectively, our study of the four putative VgrG family proteins in PCN033 reveals that only VgrG1 participates in the antibacterial ability of PCN033 and facilitates its adherence and invasion to host cells, enhancing its pathogenicity in host. Further research could focus on explore potential T6SS effectors so as to facilitate the development of effective antibacterial drugs to treat porcine ExPEC diseases.

## Materials and methods

### Strains, plasmids, and growth conditions

Strains and plasmids are listed in Table 1, and primers are presented in Table S1. All strains were grown either in Luria-Bertani (LB) broth or on LB agar plates containing 50 μg/mL ampicillin, 25 μg/mL chloramphenicol, 50 μg/mL cefotaxime, or 100 μg/mL diaminopimelic acid (Sigma, St. Louis, MO, USA).

**Table 1.** Bacterial strains and plasmids used in this study.

Strain or plasmid	Description	Source or reference
**Strain**		
PCN033	Wild-type (WT), porcine origin, O11, D Cm^S^	Lab stock
Δ*vgrG1*Δ*0248*	Mutant with gene *0247 0248* deleted in PCN033	This study
Δ*vgrG2*Δ*1588*	Mutant with gene *1587 1588* deleted in PCN033	This study
Δ*vgrG1*Δ*0248*Δ*vgrG2*Δ*1588*	Mutant with gene *0247 0248 1587 1588* deleted in PCN033	This study
Δ*vgrG1*Δ*vgrG2*Δ*1588*	Mutant with gene *0247 1587 1588* deleted in PCN033	This study
Δ*0248*Δ*vgrG2*Δ*1588*	Mutant with gene *0248 1587 1588* deleted in PCN033	This study
Δ*vgrG1*Δ*0248*Δ*vgrG2*Δ*1588/*pVgrG1	Mutant *ΔvgrG1Δ0248ΔvgrG2Δ1588* complemented with gene *vgrG*1, Cm^S^	This study
Δ*vgrG1*Δ*0248*Δ*vgrG2*Δ*1588/*p0248	Mutant*ΔvgrG1Δ0248ΔvgrG2Δ1588* complemented with gene *0248*, Cm^R^	This study
Δ*vgrG1*Δ*0248*Δ*vgrG2*Δ*1588/*pVgrG2	Mutant*ΔvgrG1Δ0248ΔvgrG2Δ1588* complemented with gene *vgrG2*, Cm^R^	This study
DH5α	F^−^,ϕ80dlacZΔM15,Δ(lacZYA-argF)U169,deoR,recA1,endA1,hsdR17 (r_k_^−^,m_k_^+^),phoA,supE44,λ^−^,thi-1,gyrA96,rel1	Takara Bio
χ7213 [88]	Thi-1 thr-1 leuB6 fhuA21 lacY1 glnV44ΔasdA4 recA1 RP4 2-Tc::Mu[λpir] Km^R^	Dr. Roy Curtiss, USA
W3110 [94]	F-, lambda- IN(rrnD-rrnE)1 rph-1, KmS	Lab stock
Δ*hcp1*Δ*hcp2*Δ*hcp3* [19]	Mutant with gene *hcp1hcp2hcp3* deleted in PCN033, Cm^S^	Lab stock
**Plasmid**		
pMD18-T	Cloning vector	Takara Bio
pSK	Shuttle vector	Lab stock
pRE112 [88]	oriT oriV Δasd Cm^R^ SacB, suicide vector	Dr. Roy Curtiss, USA
pRE112::*vgrG1 0248*	pRE112-inserted disrupted gene *vgrG1 0248*, Cm^R^	This study
pRE112::*vgrG*2 *1588*	pRE112-inserted disrupted gene *vgrG2 1588*, Cm^R^	This study
pRE112::*vgrG1*	pRE112-inserted disrupted gene *vgrG1*, Cm^R^	This study
pRE112::*vgrG2*	pRE112-inserted disrupted gene *vgrG2*, Cm^R^	This study
pHSG396 [89]	ori lacZ Cm^R^	Takara Bio
pHSG396- *vgrG1*	pHSG396-inserted gene *vgrG1*, Cm^R^	This study
pHSG396- *0248*	pHSG396-inserted gene *0248*, Cm^R^	This study
pHSG396- *vgrG2*	pHSG396-inserted gene *vgrG2*, Cm^R^	This study

### Analysis of amino acid sequences and conserved domains of VgrGs

The amino acid sequences of VgrGs were firsty aligned with BLASTP (https://blast.ncbi.nlm.nih.gov/Blast.cgi), and further analyzed with Clustal Omega (https://www.ebi.ac.uk/Tools/msa/clustalo/). The conserved domains of VgrGs were searched by their amino acid sequences on NCBI Conserved Domain Search (https://www.ncbi.nlm.nih.gov/Structure/cdd/wrpsb.cgi).

### Construction of mutant and complemented strains

Mutants were constructed by homologous recombination with the suicide plasmid pRE112, as described previously [88,89]. Sequence analysis revealed that only one amino acid was different between the sequences of VgrG1 and VgrG2, and that the sequences of 0248 and 1588 were identical (Figure S1(a)). Gene *vgrG1* was adjacent to *0248*, and *vgrG2* was adjacent to *1588* (Figure 1). The upstream homology arms of *0248* and *1588* were identical, which were the part of C-terminus of *vgrG1, vgrG2* respectively. The downstream homology arms of *vgrG1* and *vgrG2* were 99% homology, which contained *0248, 1588* respectively. Based on these, the single *vgrG* mutant is difficult to be obtained by homologous recombination (Figure S2). Therefore, mutants of the genes *vgrG10248* and *vgrG21588* were firstly constructed in PCN033 and simultaneously confirmed using internal and external source primer. On the basis of mutant Δ*vgrG2*Δ*1588*, the mutants Δ*vgrG1*Δ*vgrG2*Δ*1588*, Δ*0248*Δ*vgrG2*Δ*1588*, and Δ*vgrG1*Δ*0248*Δ*vgrG2*Δ*1588* were obtained using the same method.

The *vgrG* genes were amplified from the PCN033 genome and then ligated into plasmid pHSG396 to construct the complemented expression plasmid pHSG396-*vgrGs* [90]. And then the complemented plasmids were transferred into mutants to generate the complemented strains. All mutants and complemented strains were verified using PCR (Figure S3) and DNA sequencing (date not shown).

### Growth characteristics of mutant and complemented strains

Overnight cultures of bacteria were transferred into LB medium and grown by shaking at 180 rpm for 12 h at 37°C. The growth rates of PCN033, mutants, and complemented strains were determined with optical density measurements at 600 nm (OD_600_), and colony forming units (CFUs) were counted as described previously [91]. No growth rate difference was found when mutant and complemented strains were compared with WT strain (Figure S4(a, b)).

### Animal experiments

All animal experiments were approved by the Scientific Ethics Committee of Huazhong Agricultural University with the permit number HZAUMO-2015–027. Five-week-old female BALB/c mice were randomly divided into the groups of twelve and were intraperitoneally (i.p.) inoculated with PCN033 and mutant Δ*vgrG1*Δ*0248*Δ*vgrG2*Δ*1588* at approximate 1 × 10^6^ CFU. Mice were monitored for 336 hours (14 days) and survival rates were recorded as described in a previous study [92]. Five-week-old female BALB/c mice used for bacteria colonization were randomly divided into groups of five and were intravenously (i.v.) inoculated with PCN033, mutants, and complemented strains at approximate 1 × 10^7^ CFU (a sublethal dose of bacteria). The mice were anesthetized at 6 h post infection and their blood were collected via tail vein, then organs (brain, liver, and spleen) were harvested after the mice had been subjected to cardiac perfusion, as described in a previous study [93]. Bacterial survival in the blood is expressed as CFU/mL, and the level of bacterial multiplication capacity in the organs is expressed as CFU/g tissue.

To determine the role of VgrG*s* in inflammation, serum samples (infected with PCN033 and mutant Δ*vgrG1*Δ*0248*Δ*vgrG2*Δ*1588*) were collected, and the levels of IL-1β were measured using Mouse IL-1β Precoated ELISA kits (Catalog # DKW12-2012–096).

### Antibacterial assays

Antibacterial assays were performed as described previously with minor modifications [94]. Briefly, PCN033 and *vgrG* mutants were used as predator bacteria and a nonpathogenic *E. coli* K12 W3110 without T6SS genes was used as prey [71,95]. Bacteria were grown at 37°C, accompanied by shaking at 180 rpm to an OD_600_ = 0.8. After bacterial cultures (both predator and prey) were diluted to OD_600_ = 0.5, prey and predator were mixed at a ratio of 10:1. Finally, mixtures were spotted onto nitrocellulose membranes (Millipore, USA) on LB agar at 30°C for 12 h, and CFUs of prey were counted. Antibacterial ability was calculated according to the survival rate of W3110, which is inversely proportional to the antibacterial activity of predator.

### Adherence and invasion assays

The adherence and invasion assays were performed using confluent monolayers of HBMEC cells in 24-well plates as described previously with a few modifications [96]. Bacteria were centrifuged at OD_600_ = 0.8, and washed three times with PBS, and then resuspended in HBMEC medium (1 × 10^8^ CFU/mL) without antibiotics. One hundred microliters of bacterial suspensions were added to HBMEC cell cultures (MOI = 100). Subsequently, HBMEC cells were centrifuged at 800 g for 10 min and incubated at 37°C. After coculture for 90 min, the suspensions were removed and the cells were washed three times with PBS and then exposed to medium with cefotaxime for 1 h to kill extracellular bacteria. Finally, the cells were washed three times with PBS and lysed as described previously [97]. After a series of 10-fold dilutions, suspensions were plated on LB agar and viable bacterial cells were counted [98]. For the adherence experiments, the cefotaxime incubation process was omitted, the remaining steps were consistent with the steps of the invasion experiments. Data were expressed as the percentage of adherence and invasion relative to those of control PCN033 cultures (100%).

### Lactate dehydrogenase (LDH) activity

The cytotoxicity of bacteria to HBMEC cells was tested by lactate dehydrogenase assays [99]. Bacteria in the stationary phase resuspended in fresh medium were added into 96-well plates (MOI = 10). HBMEC cells treated with medium with or without 1% Triton X-100 served as the positive and negative controls, respectively. Relative infection ratios were then determined by using BioVision LDH-Cytotoxicity Colorimetric Assay kits (Catalog # K311-400), and LDH activities were expressed according to the following formula: Cytotoxicity (%) = (Test Sample − Low Control)/(High Control − Low Control) × 100%.

### Whole blood bactericidal experiments

Whole blood bactericidal assays were performed as previously described with minor modifications [100]. Briefly, heparinized whole blood samples were collected from jugular veins of pigs. The whole blood samples used in the same experiment was collected from one pig, and the whole blood samples used in the three whole blood bactericidal assays were collected from different pigs. Bacteria in the stationary phase were harvested and washed three times with sterile PBS and then diluted to 1 × 10^8^ CFU/mL in PBS. Subsequently, 100 μL of bacterial suspensions were mixed with 900 μL of fresh whole blood, and the mixtures were incubated at 37°C. After incubation for 3 h, bacteria were plated onto LB agar and counted. Survival rate was expressed by using the following formula: (CFU/mL)_t = 3_ _h_)/(CFU/mL)_t = 0_ _h_) × 100%.

## RNA extraction and qRT-PCR

The total RNA of bacteria were extracted using the TRIzol® reagent (Invitrogen, Paisley, UK) as reported previously [96]. And then the RNA samples were reverse transcribed to cDNA with the PrimeScript^TM^RT reagent Kit with gDNA Eraser (Takara). The transcription level of VgrG1 or VgrG2 was measured with 16s rRNA as a reference gene. The template used for measured the transcription level of VgrG1 is Δ*vgrG2*Δ*1588* and the template used for measured the transcription level of VgrG2 is Δ*vgrG1*Δ*0248*. PCN033 and Δ*vgrG1*Δ*0248*Δ*vgrG2*Δ*1588* used as the positive and negative controls, respectively. Primers used for the qPCR are listed in Table S1.

## Statistical analysis

All assays were performed in triplicate at three different time, and the data were analyzed using GraphPad Prism 6 (2) (San Diego, CA, USA). Survival rate was analyzed by the log-rank test. The number of bacteria in mice tissues was compared by Tukey post hoc test. The multiple comparisons (Figure 2(c), 4, 5, 6) were analyzed by ANOVA. *P* < 0.05 was considered as significant difference.

## Ethics statement

This study was carried out in strict accordance with the Guide for the Care and Use of Laboratory Animals Monitoring Committee of Hubei Province, China. The protocols and procedures were approved by the Committee on the Ethics of Animal Experiments at the College of Huazhong Agricultural University (permit number: HZAUMO-2015–027).
