# Supplementary material for: Characterization of multiple type-VI secretion system (T6SS) VgrG proteins in the pathogenicity and antibacterial activity of porcine extra-intestinal pathogenic Escherichia coli
Source: Virulence. 2019 Jan 24;10(1):118–32. doi: 10.1080/21505594.2019.1573491 (PMC6363058; doi:10.1080/21505594.2019.1573491)
Supplement: Supplemental Material [file kvir-10-01-1573491-s001.doc]

**Supplementary Table 1. List of oligonucleotide primers used in this study**

| Primer | Sequence(5'-3') | Remark |
| --- | --- | --- |
| 12U_F_ | CGG**GGTACC**TCATGATTCAGCTTTGGCC (*Kpn*Ⅰ) | upstream flanking of *vgrG10248* (880bp) |
| 12U_R_ | CCG**CTCGAG**TGAACGTAACCCCTTACTGAC (*Xho*Ⅰ) |
| 12D_F_ | CCG**CTCGAG**TCCCGTACCTGTTCGGTAT (*Xho*Ⅰ) | downstream flanking of *vgrG10248* (880bp) |
| 12D_R_ | C**GAGCTC**TGTCAGGGGAAAATAAGGAA (*Sac*Ⅰ) |
| 34U_F_ | CGG**GGTACC**TGCGATTGCTGATGCTGT (*Kpn*Ⅰ) | upstream flanking of *vgrG21588* (1180bp) |
| 34U_R_ | C**GAGCTC**AGCAACCTCCGTGAAAATG (*Sac*Ⅰ) |
| 34D_F_ | C**GAGCTC**GGTAAGGAGCAGGTTAATGGTG (*Sac*Ⅰ) | downstream flanking of *vgrG21588* (1100bp)  upstream flanking of  *vgrG*1 (1000bp)  downstream flanking of *vgrG*1 (990bp)  upstream flanking of  *vgrG*2 (616bp)  downstream flanking of *vgrG*2 (634bp)  amplification gene *vgrG*1(1626bp)  amplification gene *vgrG*2(477bp) |
| 34D_R_  1U_F_  1U_R_  1D_F_  1D_R_  2U_F_  2U_R_  2D_F_  2D_R_  V1_F_  V1_R_  V2_F_  V2_R_ | TAG**TCTAGA**GGGAACGGTCAGAAGAAGATAG (*Xba*Ⅰ)  CGG**GGTACC**TCATGATTCAGCTTTGGCC (*Kpn*Ⅰ)  C**GAGCTC**AGCAACCTCCGGGTTTAAG (*Sac*Ⅰ)  C**GAGCTC**AGACGGTGGGCAGTAACC (*Sac*Ⅰ)  CTAG**TCTAGA**CTGCCTTACCACCGCG (*Xba*Ⅰ)  CGG**GGTACC**CCACGCTGAATAACCACTTT (*Kpn*Ⅰ)  C**GAGCTC**CGTCTCTATCTGGTTAACCCC (*Sac*Ⅰ)  C**GAGCTC**TCCCGTACCTGTTCGGTATTCA (*Sac*Ⅰ)  CTAG**TCTAGA**CTGACCACCCCAGTATCCACC (*Xba*Ⅰ)  CCG**CTCGAG**ATGTCAACCGGATTACGTTTC (*Xho*Ⅰ)  CCG**GAATTC**CTATCTGGTTAACCCCACCG (*EcoR*Ⅰ)  CCG**CTCGAG**GTGGGCAGTAACCAGATCATT (*Xho*Ⅰ)  CCG**GAATTC**TCAGTACTCTCTCATATCCGGC (*EcoR*Ⅰ) |
| P1 | CAGCGAACTGTCTGTTTTTC | internal source primers  for ∆*vgrG1*∆*0248* |
| P2 | GGCTGCTCGCCGTG |
| P3 | AAATGCTGCCAACCACTG | external source primers  for ∆*vgrG1*∆*0248* |
| P4 | GCGGCTCAAGGTGAATG |
| P5 | AAATCCCAGACTAAATCATCACA | internal source primers  for ∆*vgrG2*∆*1588* |
| P6 | CAAAGGACTCCGGCAGATA |
| P7 | ATCCCAGACTAAATCATCACATAA | external source primers |
| P8  P9  P10  P11  P12  P13  P14  P15  P16  P17  P18  16SrRNA_F_  16SrRNA_R_  VgrG_F_  VgrG_R_ | GTCCGTCATCACGTAGCTGT  GACACCGTGCGTTATCTGC  TCTGCCGGTATTACCGCA  TGTACGAGCCTGTCGTGAA  TAAATCGCGGTCTGCC  TATCAGACGACGGTAGGTGG  GTCACCATTAACCTGCTCCTT  CGGGTTTAATGAACTGAAGTTT  CGGATTTACCGGGTTGC  GCTCACTCATTAGGCACCC  GCTCATTCGCCATTCAGG  GAATGCCACGGTGAATAC  GGTTACCTTGTTACGACTTC  TCATTGTGGACTTCCTCAA  CGTGGATGTAGACCTGTT | for ∆*vgrG2*∆*1588*  internal source primers  for ∆*vgrG1*∆*vgrG2*∆*1588*  external source primers  for ∆*vgrG1*∆*vgrG2*∆*1588*  internal source primers  for ∆*0248*∆*vgrG2*∆*1588*  internal source primers  for ∆*0248*∆*vgrG2*∆*1588*  universal primers of plasmid pHSG396(249bp)  quantitative primer for  16SrRNA (150bp)  quantitative primer for  VgrG1 and VgrG2 (189bp) |

Note: Sequence labeling “-” means Restriction Enzyme cutting site.

**Supplementary table 2. The potential effectors of T6SS in PCN033**

| **Locus number** | **Predited function** | | | **Length (bp)** |
| --- | --- | --- | --- | --- |
| PCN033_0229 |  |  | T6SS associated protein | 744 |
| PCN033_0236 |  |  | Transposase IS200 group | 459 |
| PCN033_0240 |  | T6SS lysozyme-like protein | | 129 |
| PCN033_0242 |  | Hypothetical protein | | 207 |
| PCN033_0244 |  | Hypothetical protein | | 129 |
| PCN033_0246 |  | Hypothetical protein | | 138 |
| PCN033_0249 |  | RHS protein contains YD-repeat regions | | 4263 |
| PCN033_0251 |  | RHS protein contains YD-repeat regions | | 1575 |
| PCN033_0252 |  | RHS repeat-associated core domain | | 2277 |
| PCN033_0255 | Transposase DDE domain | | | 1137 |
| PCN033_0256 | Predicted amidohydrolase (nitrilase family) | | | 771 |
| PCN033_0258 |  | acyl-CoA dehydrogenase | | 2445 |
| PCN033_0259 |  | Phosphoheptose isomerase | | 579 |
| PCN033_0260 |  | Glutamine amidotransferases class-II | | 768 |
| PCN033_0261 |  | Murein L,D-transpeptidase | | 741 |
| PCN033_0262 |  | NlpC/P60 family; It is found in several lipoproteins | | 759 |

**
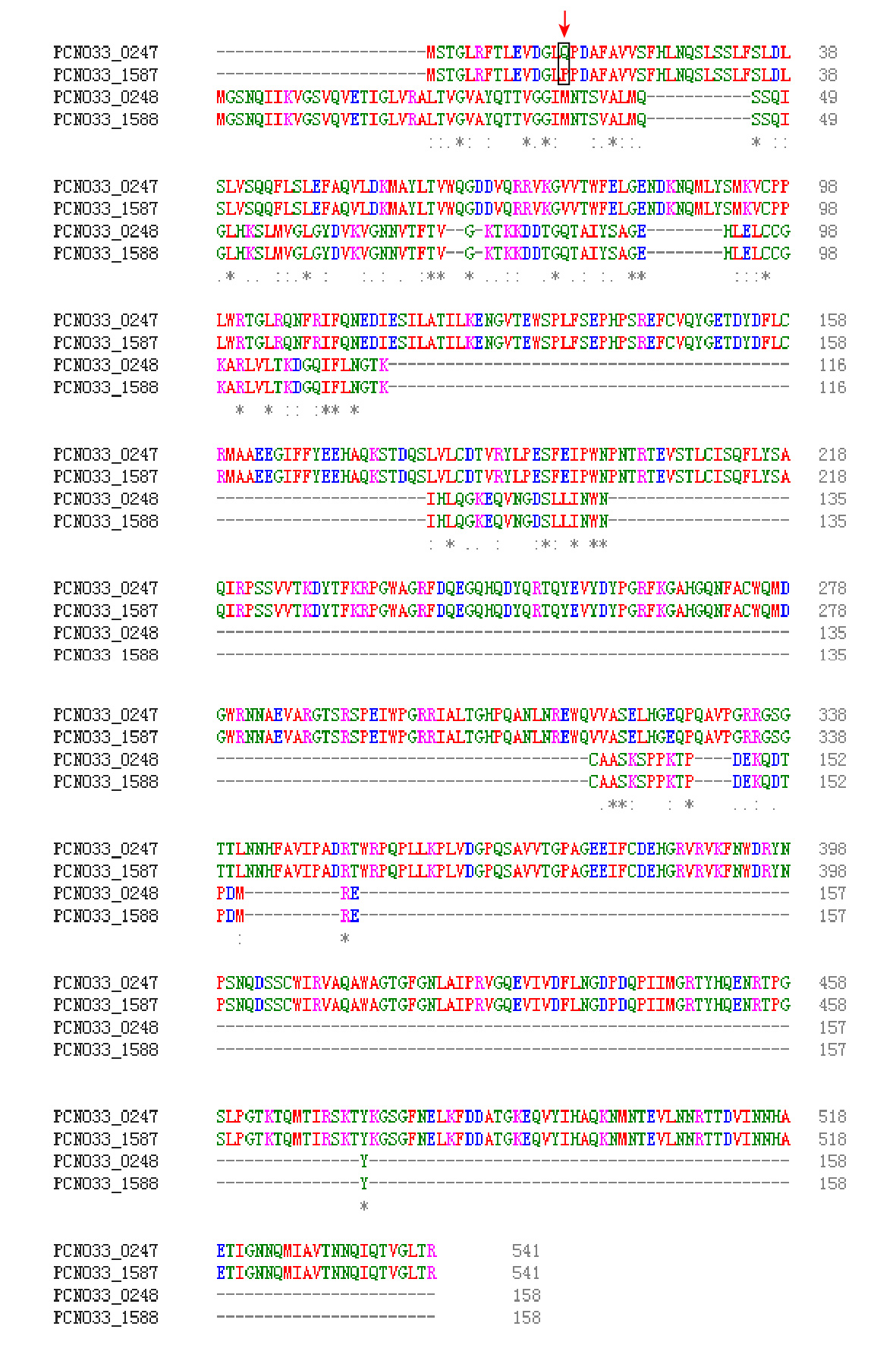
**

**Supplementary Figure 1A.** **Amino acid sequence alignment of PCN033 VgrGs.** The amino acid sequences of PCN033_0247 and PCN033_1587 are 99% homologous, actually they only have one different amino acid. The different amino acid is marked with a black box and indicated by a red arrow. The amino acid sequences of PCN033_0248 and PCN033_1588 are 100% homologous.

**
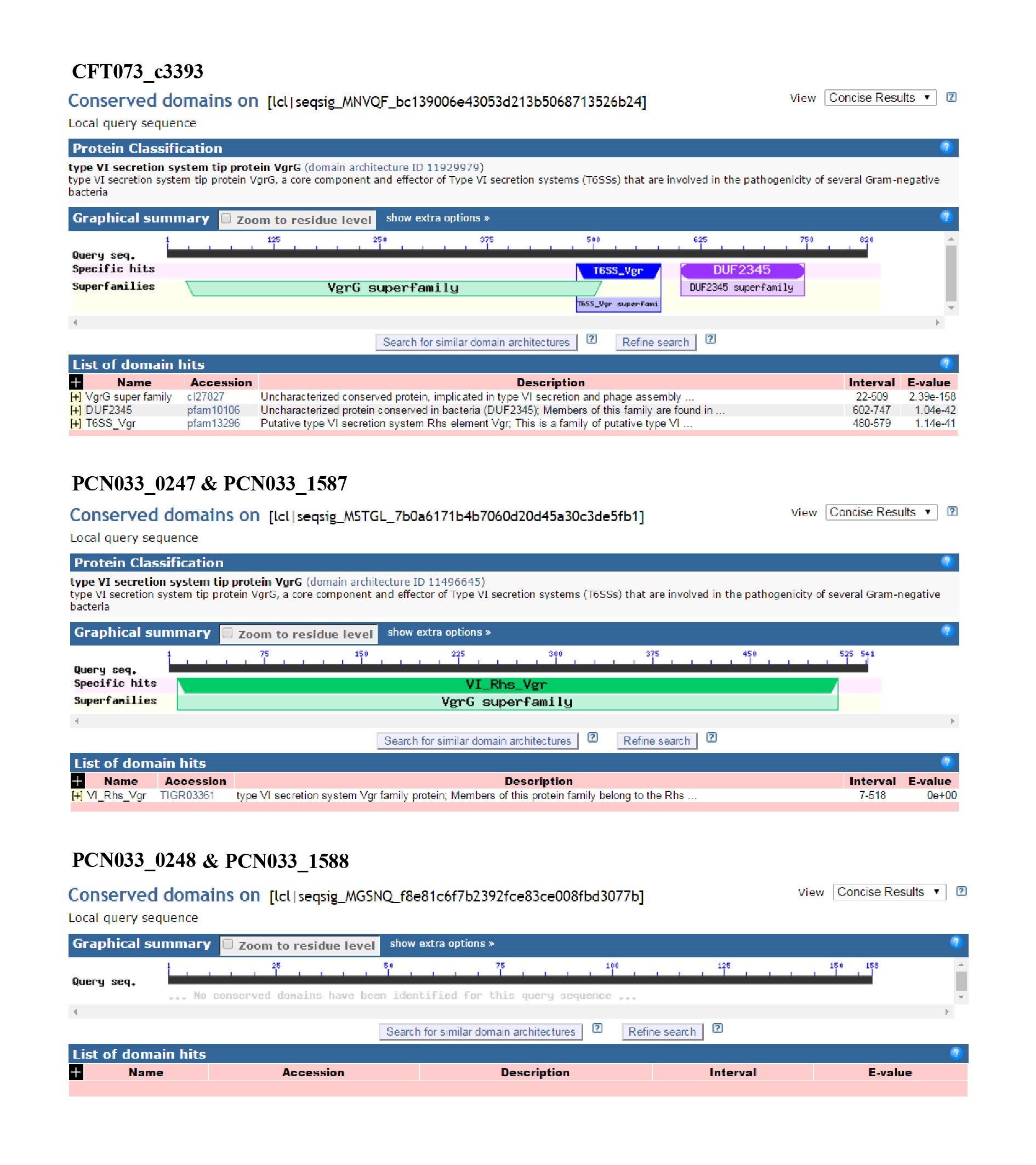
Supplementary Figure 1B.** **Conserved domain of PCN033 VgrGs and CFT073 VgrG.** CFT073 VgrG possesses the VgrG domain and a DUF2345 domain. PCN033_0247 and PCN033_1587 possess the conserved VgrG domain. PCN033_0248 and PCN033_1588 have no conserved domain.

**
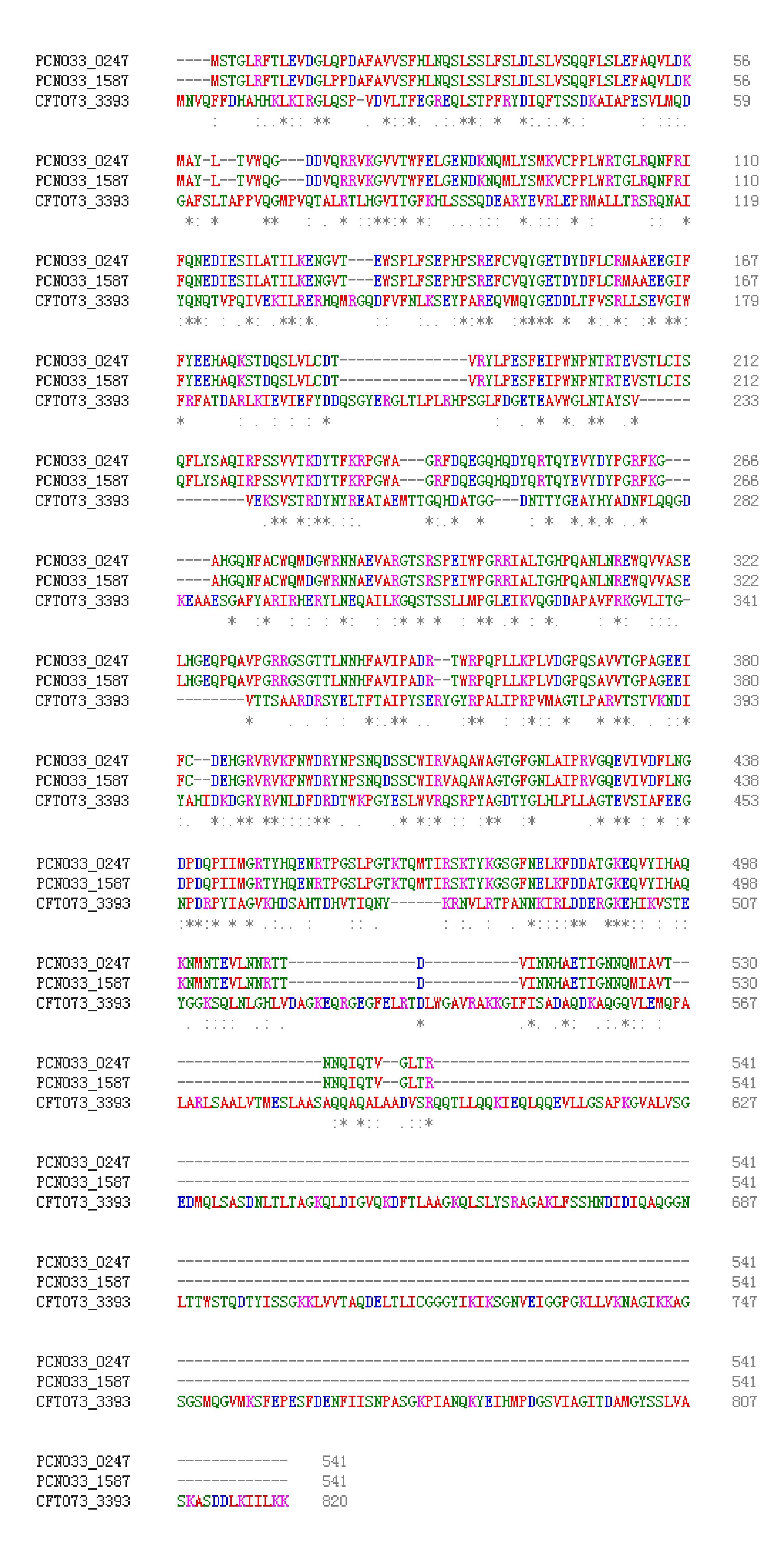
**

**Supplementary Figure 1C.** **Amino acid sequence alignment of PCN033_0247, PCN033_1587, and CFT073_c3393.** **PCN033_0247 and PCN033_1587,** has about 25% homology with the N-terminus (residues 1-597) of CFT073_c3393.


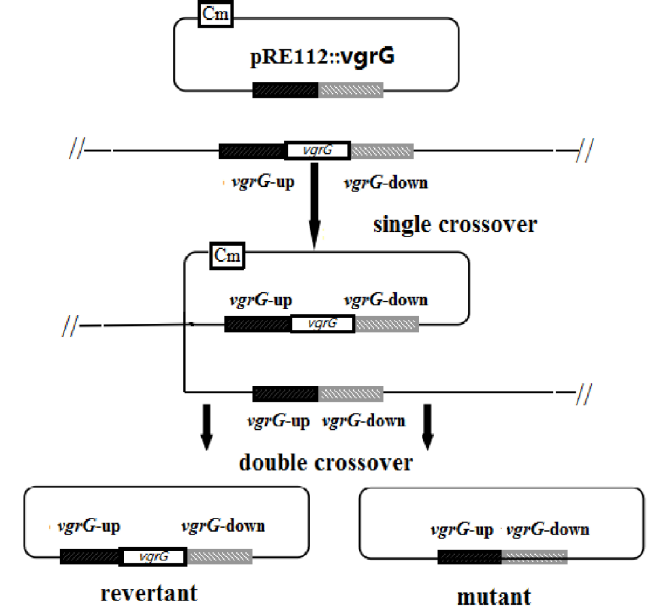


**Supplementary Figure 2.** **The schematic diagram of the homologous recombination method.** Markerless chromosomal *vgrG* gene deletion mutants were constructed in porcine ExPEC strain PCN033 by homologous recombination.


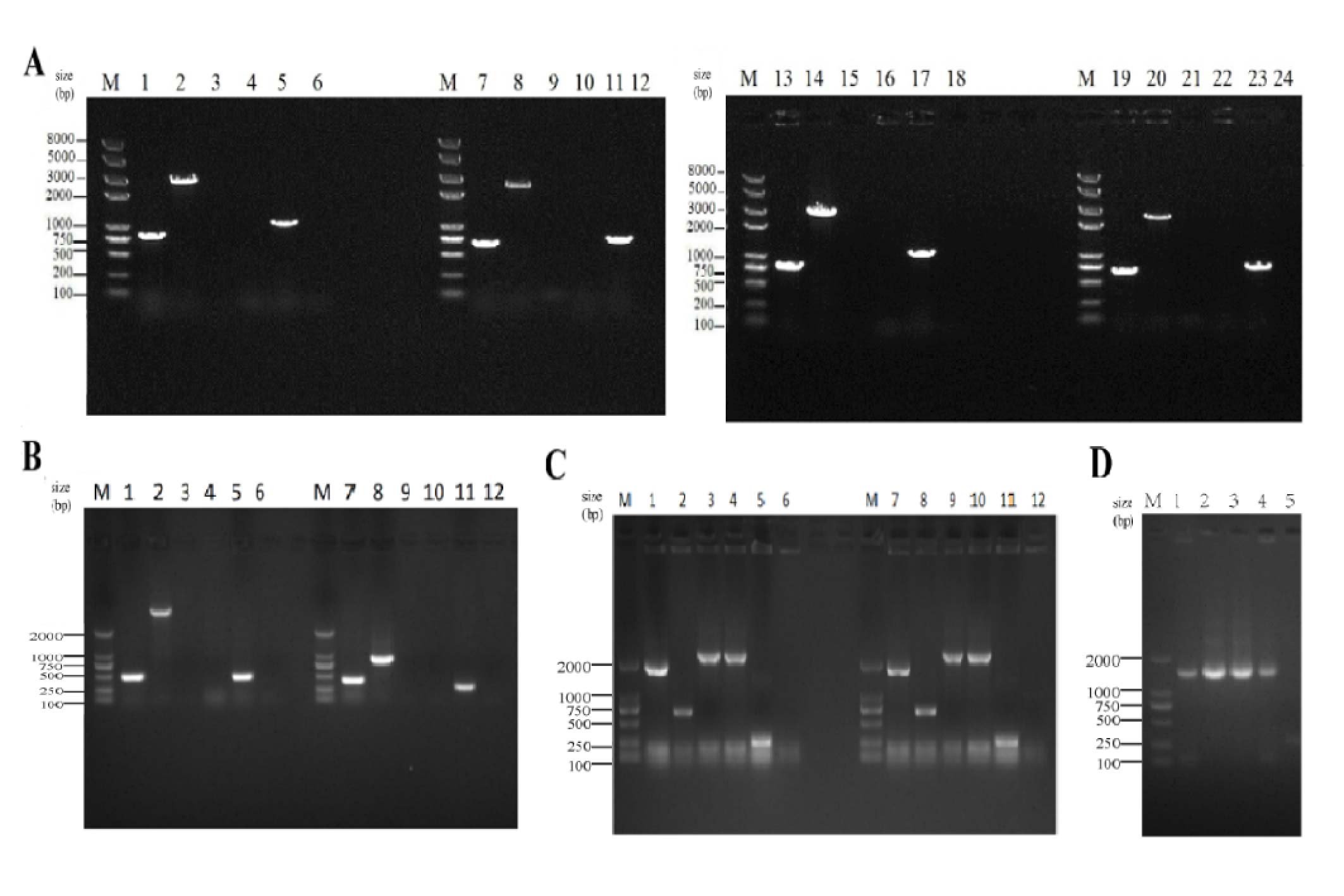


**Supplementary Figure 3.** **Construction of mutant and complemented strains.** The sequences of primer pairs used in the PCR analysis are presented in **Table S1**. (A) Construction of mutants *∆vgrG1*Δ*0248*, *∆vgrG2*Δ*1588*, *∆vgrG1*Δ*0248*Δ*vgrG2*Δ*1588*. M: DNA markers (DL8000) 1: ∆*vgrG1*∆*0248* (P3/P4); 2: PCN033 (P3/P4); 3: negative control (P3/P4); 4: ∆*vgrG1*∆*0248* (P1/P2); 5: PCN033 (P1/P2); 6: negative control (P1/P2); 7: ∆*vgrG2*∆*1588* (P7/P8); 8: PCN033 (P7/P8); 9: negative control (P7/P8); 10: ∆*vgrG2*∆*1588* (P5/P6); 11: PCN033 (P5/P6); 12: negative control (P5/P6); 13: *∆vgrG1*Δ*0248*Δ*vgrG2*Δ*1588* (P3/P4); 14: PCN033 (P3/P4); 15: negative control (P3/P4); 16: *∆vgrG1*Δ*0248*Δ*vgrG2*Δ*1588* (P1/P2); 17: PCN033 (P1/P2); 18: negative control (P1/P2); 19: *∆vgrG1*Δ*0248*Δ*vgrG2*Δ*1588* (P7/P8); 20: PCN033 (P7/P8); 21: negative control (P7/P8); 22: *∆vgrG1*Δ*0248*Δ*vgrG2*Δ*1588* (P5/P6); 23: PCN033 (P5/P6); 24: negative control (P5/P6). (B) Construction of mutants *∆vgrG1*Δ*vgrG2*Δ*1588* and Δ*0248*Δ*vgrG2*Δ*1588*. M: DNA markers (DL2000) 1: *∆vgrG1*Δ*vgrG2*Δ*1588* (P11/P12); 2: PCN033 (P11/P12); 3: negative control (P11/P12); 4: *∆vgrG1*Δ*vgrG2*Δ*1588* (P9/P10); 5: PCN033 (P9/P10); 6: negative control (P9/P10); 7: Δ*0248*Δ*vgrG2*Δ*1588* (P15/P16); 8: PCN033(P15/P16); 9: negative control (P15/P16); 10: Δ*0248*Δ*vgrG2*Δ*1588* (P13/P14); 11: PCN033 (P13/P14); 12: negative control (P13/P14). (C) Construction of the corresponding complemented strains. M: DNA markers (DL2000) 1: ∆*vgrG1*∆*0248*/pVgrG1 (P17/P18); 2: *∆vgrG1*Δ*0248*/p0248 (P17/P18); 3: *∆vgrG1*Δ*0248*/pVgrG1p0248 (P17/P18); 4: *∆vgrG1*Δ*0248*/pVgrG1p0248(P17/P18); 5: *∆vgrG1*Δ*0248*/pHSG396 (P17/P18); 6: negative control (P17/P18); 7: ∆*vgrG1*∆*0248*∆*vgrG2*∆*1588*/pVgrG1 (P17/P18); 8: ∆*vgrG1*∆*0248*∆*vgrG*2∆*1588*/p0248 (P17/P18); 9: ∆*vgrG1*∆*0248*∆*vgrG2*∆*1588*/pVgrG1p0248 (P17/P18); 10: ∆*vgrG1*∆*0248*∆*vgrG2*∆*1588*/pVgrG2p1588 (P17/P18); 11: ∆*vgrG1*∆*0248*∆*vgrG2*∆*1588*/pHSG396 (P17/P18); 12: negative control ((P17/P18). (D) Construction of complemented strains ∆*vgrG1*∆*0248*∆*vgrG2*∆*1588*/pVgrG2. Gene *vgrG2* were amplified from mutant Δ*vgrG1*Δ*0248* using primer pairs V1_F_/V1_R_. M: DNA markers (DL2000) 1-4: ∆*vgrG1*∆*0248*∆*vgrG2*∆*1588*/pVgrG2 (P17/P18); 5: negative control (P17/P18).


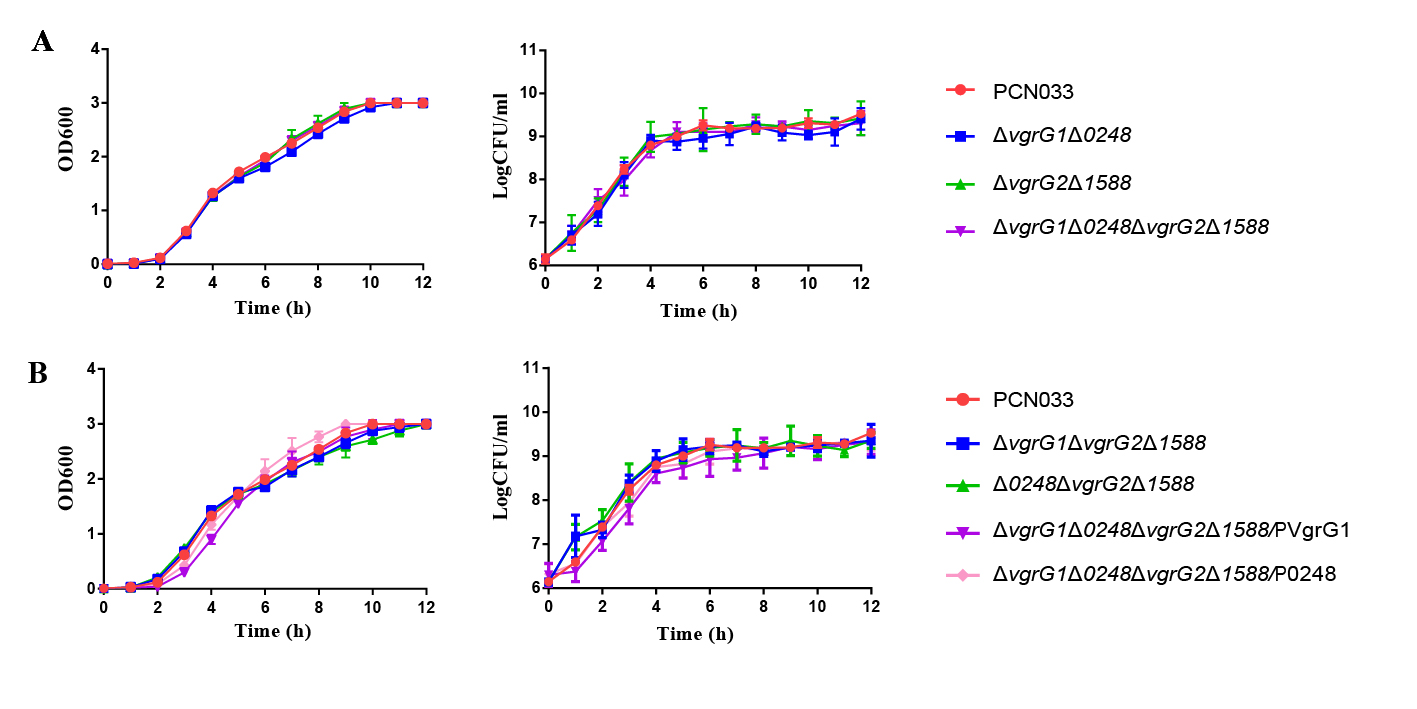


**Supplementary Figure 4.** **Growth characteristics of** **PCN033, mutants and complemented strains.** (A) OD_600_ and CFU per ml of parental and mutant strains during 12 h generating time; (B) OD_600_ and CFU per ml of parental, mutants and complemented strains during 12 h generating time.


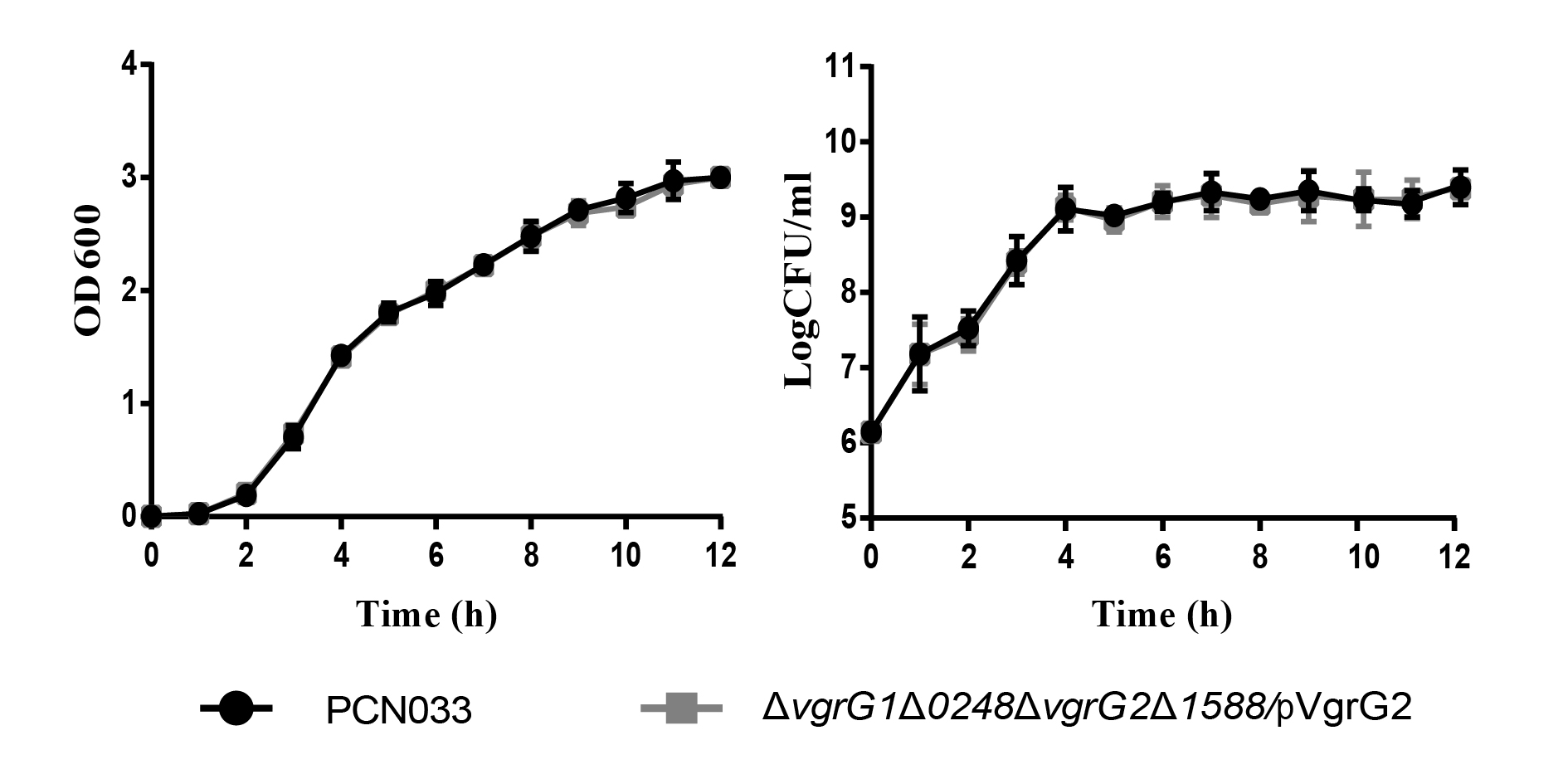


**Supplementary Figure 5.** **Growth characteristics of PCN033, mutants and complemented strains.** OD_600_ and CFU per ml of parental and complemented strain Δ*vgrG1*Δ*0248*Δ*vgrG2*Δ*1588*/pVgrG2 during 12 h generating time.
